# Hospital for the Insane in British Columbia

**Published:** 1904-10-29

**Authors:** 


### HOSPITAL FOR THE INSANE IN BRITISH COLUMBIA.

This hospital has one of the prettiest situations occupied by any similiar institution, overlooking as it does the well-known Fraser River, backed by the snow-capped Selkirks. This unique position with its scenic effects has doubtless helped to calm many a troubled mind, and to assure many a prisoner, in spite of his present chequered existence owing to mental affliction, of the love of the All-Father. Here the able Superintendent, Dr. Manchester, is doing his level best to solve the problem—how to treat patients on the Christian humanitarian lines, which he considers are the only right and practical ones, with very limited funds and difficulties immeasurable caused by mistakes and ignorance in the past.

How successfully he has raised the general condition and the individual treatment of patients is only known by those who were aware of the existing state of things when he took the helm four years ago.

Instead of trestles and boards with iron spoons and enamelled plates and cups, the patients sit down to well-laid tables, with a dietary both varied and plentiful, which would put many of our English county asylums to shame.

The diet-sheet is changed every fortnight, but the following will give an approximate idea, copied from that hanging up in the kitchen.

Breakfast: Mush (oatmeal or corn-meal); Tea or coffee; Bread and butter; Stewed fruit or maple syrup.

Dinner: Two or three courses daily; Soup or fish; Meat with two vegetables; Pudding.

Tea: Bread and butter ad lib.; Jam or stewed fruit; Meat for the workers; Tea.

The quantity is not limited, and the patient's hunger is always satisfied.

One striking feature is the open-ward system. As soon as possible patients are put on parole, and the benefit to patients (and attendants also) is very apparent and it is very rarely that any abuse of this confidence is made. In the men's open-ward there is a small flame allowed for the use of smokers. At the villa, where the female patients who are more or less convalescent are housed in single or double-bedded rooms, here they may keep their own small personal possessions, and those who wish can help in the ordinary household work.

In addition to his ordinary official visits, the superintendent who personally inspects all the work carried on, pays constant informal visits, so that any harshness or negligence is not likely to escape his eagle eye. Any patient wishing to have a personal interview with him can do so, and any complaints are always carefully investigated and every precaution taken to prevent any revenge being taken for making such a complaint.

The fact that only four to five sedatives are given on an average daily, and that no use is made of the padded-room, is sufficient testimony to the admirable manner in which the institution is worked, receiving as it does the criminal insane and many foreigners of somewhat desperate character.

The only form of partial restraint used is a kind of tunic-jacket, laced at the back, the sleeves being made very long and gradually narrowed, till at the bottom they end in bands which tie round the waist, thus confining the upper limbs, which are usually the offending members, to the side. This "camisole," as it is called, is made of stout 10-oz. duck, is worn over the ordinary clothing by both male and female patients, and is found sufficient to restrain the movements of the acutely insane.

The accommodation is really inadequate for the 380 patients, its normal capacity being 300, and the available ground amounts to only 30 acres, but a section of 1,000 acres on the Fraser has now been acquired and a farm colony will soon be established, which will relieve the congested condition of the hospital, and will supply some of its material needs. There is a nice little operating-room with an adjoining ward, where aseptic principles can be carried out.

The modern views which seek the causes of mental disease in physical derangement are followed up, often with great success. Disease of the pelvic organs, surgically treated, has often restored the mental balance, which had been upset by nerve irritation from internal organs.

The attendants are well trained, obtained mostly from other asylums. The general tone of the rules issued is gathered from the introductory remarks:—"The simplest of rules is also the best."

"Do as you would be done by," which is laid down as the basis of their behaviour towards the patients and their fellow workers. They are forcibly reminded that the hospital is built and maintained for the care and cure of those who are sick in mind (and often in body too) and that it exists for no other purpose. "Though the institution and all its resources belong to the patients, yet by reason of disordered minds they are largely without power to assert their rights, and are dependent therefore upon our sense of honour and self-respect."

The duties of the superintendent and every official are distinctly laid down in the rule-book.

The three watchwords with reference to treatment of the patients are safety, kindness, employment. The following are absolutely forbidden and are regarded as sufficient causes for discharge.

Tripping a patient, placing the weight of the body or knee on the chest or abdomen, striking a patient, twisting arms or wrists, or holding by the hair. Also patients may not be forced to take food, except in the presence of the physician or head attendant. From these extracts it is seen that the patients and their interests are paramount and that everything is done to secure their recovery or amelioration.

Dr. Manchester was most kind in personally conducting me over the hospital and in answering all my questions. The general harmony and quiet that prevailed were very striking in an institution where jarring elements seem inevitable.

The percentage of insane in British Columbia is the least of any Canadian province, being only 1·67 per 1,000 inhabitants, but a large number of those committed have come from foreign countries and other parts of Canada. Dawson contributes a goodly number, and it is noticeable how many of those who are compelled to live a very isolated life fall victims to mental disease. The Asylum is situated in the town of New Westminster, which may be reached by steamer or train and by electric car from Vancouver.

